# Gelsemin

**Published:** 1860-05

**Authors:** 


*Gelsemin.*—In the "Medical Press," for January 2d, Dr. B. Keith, of New York, has the following upon gelsemin; which article he says he has used daily for the last eight years: "For controling fevers of every type and grade; to arrest hœmorrhage from the lungs, stomach, bowels, uterus, and urinary organs; in dysentery and bowel complaints; in spermatorrhœa, amaurosis, deafness, catarrhal affections, and hay fever, I have used the gelsemin successfully. A single half grain has arrested hœmorrhage from the lungs, when all other remedies known to me had failed. While experimenting with it to ascertain its power for arresting hœmorrhage, I gave to a lady who had been confined two days previous, one and a half grains during twenty-four hours, which amount completely arrested the hœmorrhage. I administered two grains, during the course of thirty-six hours, to a lady who had been suffering from uterine hœmorrhage for two months, and that small quantity completely stopped the flow. So effectual is it in this form of hœmorrhage, that I consider it quite a specific. In dysentary and bowel complaints, I consider it the most valuable article in the Materia Medica. From one-tenth to one-eighth of one grain administered after each discharge, will shortly stop all hæmorrhage and traces of the disease." * * * "In dry coughs, depend upon iritation of the throat, it is the most prompt agent I have ever used. In nausea and vomiting I have used it, many cases yielding to a single dose of one-fourth of one grain."

